# Role of GLUT1 in regulation of reactive oxygen species

**DOI:** 10.1016/j.redox.2014.03.004

**Published:** 2014-03-25

**Authors:** Stanley Andrisse, Rikki M. Koehler, Joseph E. Chen, Gaytri D. Patel, Vivek R. Vallurupalli, Benjamin A. Ratliff, Daniel E. Warren, Jonathan S. Fisher

**Affiliations:** Department of Biology, Saint Louis University, 3507 Laclede Ave, St. Louis, MO 63103, USA

**Keywords:** Ataxia telangiectasia mutated (ATM), Oxidative stress, Insulin resistance, Glucose transport, Antioxidant

## Abstract

In skeletal muscle cells, GLUT1 is responsible for a large portion of basal uptake of glucose and dehydroascorbic acid, both of which play roles in antioxidant defense. We hypothesized that conditions that would decrease GLUT1-mediated transport would cause increased reactive oxygen species (ROS) levels in L6 myoblasts, while conditions that would increase GLUT1-mediated transport would result in decreased ROS levels. We found that the GLUT1 inhibitors fasentin and phloretin increased the ROS levels induced by antimycin A and the superoxide generator pyrogallol. However, indinavir, which inhibits GLUT4 but not GLUT1, had no effect on ROS levels. Ataxia telangiectasia mutated (ATM) inhibitors and activators, previously shown to inhibit and augment GLUT1-mediated transport, increased and decreased ROS levels, respectively. Mutation of an ATM target site on GLUT1 (GLUT1-S490A) increased ROS levels and prevented the ROS-lowering effect of the ATM activator doxorubicin. In contrast, expression of GLUT1-S490D lowered ROS levels during challenge with pyrogallol, prevented an increase in ROS when ATM was inhibited, and prevented the pyrogallol-induced decrease in insulin signaling and insulin-stimulated glucose transport. Taken together, the data suggest that GLUT1 plays a role in regulation of ROS and could contribute to maintenance of insulin action in the presence of ROS.

## Highlights

• Inhibition of GLUT1, but not inhibition of GLUT4, increases ROS levels in myoblasts.
• Mutation of an ATM target site on GLUT1 to alanine (GLUT1-S490A) increases ROS.
• The ATM activator doxorubicin decreases ROS except in cells that express GLUT1-S490A.
• Inhibition of ATM increases ROS except in cells transfected with GLUT1S490D.
• Expression of GLUT1-S490D protects cells against ROS-mediated insulin resistance.

## Introduction

Reactive oxygen species (ROS) are a byproduct of metabolism, being produced, for example, during electron transfer in mitochondrial respiration [1]. ROS can serve as signaling molecules for metabolic processes including PI3K- and AMPK-independent glucose transport [2], PGCα-stimulated mitochondrial biogenesis [3], and regulation of muscle-derived cytokines [4]. However, prolonged oxidative stress, as apparent in obesity and metabolic syndrome, is associated with an elevation in reactive oxygen species (ROS) [5]. Elevated levels of ROS have been shown to be associated with insulin resistance [6], a key feature of type 2 diabetes mellitus [7]. For example, mice fed a high-fat diet to induce insulin resistance displayed increased ROS levels in adipocytes and skeletal muscle [8]. Intriguingly, treatment with an antioxidant prevented insulin resistance caused by a high fat diet [8].

Glucose transporter 1 (GLUT1) transports glucose [9] and dehydroascorbic acid (DHA) [10], the oxidized form of ascorbate. Both glucose and ascorbic acid have roles in antioxidant defense. Glucose, in the form of glucose 6-phosphate, is the first substrate in the pentose phosphate pathway, which generates the electron carrier NADPH [11]. NADPH donates electrons to antioxidant pathways such as those including glutathione reductase, thioredoxin reductase, or reduction of DHA to ascorbate, and these pathways in turn depress ROS levels [12,13]. Previous work has shown that increased activity of ataxia telangiectasia mutated (ATM) leads to increased GLUT1-mediated glucose and DHA transport in a mechanism that likely involves phosphorylation of GLUT1 at serine 490 (S490) [14]. Thus, it seems possible that ATM, which acts as a ROS sensor and activates glucose 6-phosphate dehydrogenase (G6PDH) in response to oxidative stress, could also promote antioxidant defense through regulation of GLUT1.

The objective of the present study was to determine whether GLUT1 contributes to regulation of ROS levels in myoblasts. Here we show that inhibition of GLUT1, but not GLUT4, increases ROS. Additionally, activation of ATM decreases ROS, except in cells expressing a GLUT1 serine•alanine mutation at an ATM target site, S490. Further, inhibition of ATM increases ROS, except in cells expressing GLUT1 with an ATM target site mutated to aspartate (S490D). Finally, we found that expression of GLUT1-S490D protects against ROS-induced insulin resistance.

## Materials and methods

### Materials

Phosphate buffered saline (PBS), trypsin, Dulbecco tm)s modified Eagle tm)s medium (DMEM), antimycin A, phloretin, and fasentin were acquired from Sigma Aldrich (St. Louis, MO, USA). The general ROS and superoxide specific fluorescent probes, CM-H2DCFDA (DCFDA) and Mitosox Red, respectively, were purchased from Life Technologies, Invitrogen (Eugene, OR, USA). Doxorubicin was purchased from Sigma-Aldrich (St. Louis, MO, USA). The ATM inhibitor KU55933 (KU) [15] was a gift from KuDos Pharmaceuticals, Ltd. (Cambridge, UK). A second ATM inhibitor, CP466722 (CP) [16], was obtained from Pfizer (Groton, CT). Antibodies against phosphorylated Akt S473 and Akt were obtained from Cell Signaling Technology (Danvers, MA, USA). Antibodies against GAPDH (conjugated to horseradish peroxidase) and phosphorylated rat/mouse IRS-1 Y612 (equivalent to Y608 in murine IRS-1) were purchased from Sigma-Aldrich (St. Louis, MO, USA).

### Cell culture

L6 myoblasts were bought from the American Type Culture Collection (Manassas, VA). DMEM containing 10% FetalPlex (Gemini Bio-Products, Woodland, CA) and 1% antibiotic•antimycotic solution were used to culture the L6 myoblasts at 37 °C in 5% CO_2_.

### GLUT1 constructs and transfections

The FLAG-GLUT1 construct [17], containing an exofacial FLAG epitope in the first extracellular loop, was kindly contributed by Jeffrey Rathmell (Duke University). Site mutants, GLUT1-S490A and GLUT1-S490D, were generated as described previously [14]. Compared to myotubes, L6 myoblasts display depressed GLUT3 expression [18]. Thus, we used myoblasts instead of myotubes to minimize any potential role of GLUT3, a low affinity glucose and DHA transporter, in the outcome measures.

### Reactive oxygen species assays

L6 myoblasts were loaded with fluorescent probes (primarily 10 α/4M DCFDA but for one experiment 5 α/4M Mitosox Red in serum free DMEM) for 30•60 min, washed with PBS to clear away phenol red, and incubated in HEPES buffered saline (HBS: 5 mM glucose, 20 mM HEPES, 140 mM NaCl, 5 mM KCl, 2.5 mM MgSO_4_, and 1 mM CaCl_2_) to obtain a baseline fluorescence reading. DCFDA has an excitation/emission of 485/528 nm. MitoSox Red has an excitation/emission of 485/590 nm. DCFDA is a general ROS probe [19]. MitoSox Red is a fluorogenic dye that accumulates in mitochondria and purportedly detects superoxide anions [20]. After the baseline reading, the L6 myoblasts were incubated in serum-free media plus and minus the appropriate treatment, then a second fluorescence reading was taken followed immediately by exposure to ROS inducers, antimycin A (Ant-A) and pyrogallol (PG). Ant-A stimulates mitochondrial superoxide production via disruption of cytochrome *c* reductase in the electron transport chain [21]. Pyrogallol generates superoxide by nonenzymatic reaction with oxygen [22]. Finally, a kinetic fluorescence reading was taken. A portion of the plates contained no cells or non-loaded cells as negative controls.

### Insulin action experiments

L6 myoblasts transiently transfected with FLAG-GLUT1 constructs were serum starved in HBS for 2 h and then treated plus or minus 100 nM insulin and 50 α/4M pyrogallol, a ROS generator, for 1 h. Subsequently, the cells were washed twice with phosphate buffered saline (PBS) then subjected to either a Western blot analysis or glucose transport assay (both described below).

### Western blot analysis

L6 myoblasts transiently transfected with FLAG-GLUT1 constructs and incubated as explained above were subjected to Western blot analysis as described previously [14].

### Transport assays for cultured myoblasts

L6 myoblasts, treated as explained in insulin action experiments above, were exposed to 2-deoxy-glucose (2DG) transport media (0.5 α/4Ci/ml 3H-labeled 2DG, 10 α/4M 2DG, dissolved in glucose-free HBS) for 10 min. An ice-cold 0.9% saline solution was used to wash the cells. The cells were then incubated with lysis buffer (0.2% SDS and 0.2 N NaOH) for 30 min and processed for scintillation counting as described previously [14]. A BCA assay was performed using the samples to determine protein content. To account for non-specific 2DG uptake, a subgroup of the cells was treated in the presence of 10 α/4M cytochalasin B. Cytochalasin B prevents transport by GLUTs [23].

### Statistical analysis

Univariate one-way analysis of variance (ANOVA) with a significance determined as *p* < 0.05 was used to analyze the data. The software used was SPSS Statistics 21.0 (IBM, Armonk, NY, USA).

## Results

### Inhibition of GLUT1 elevates ROS levels

**Fig. 1:**
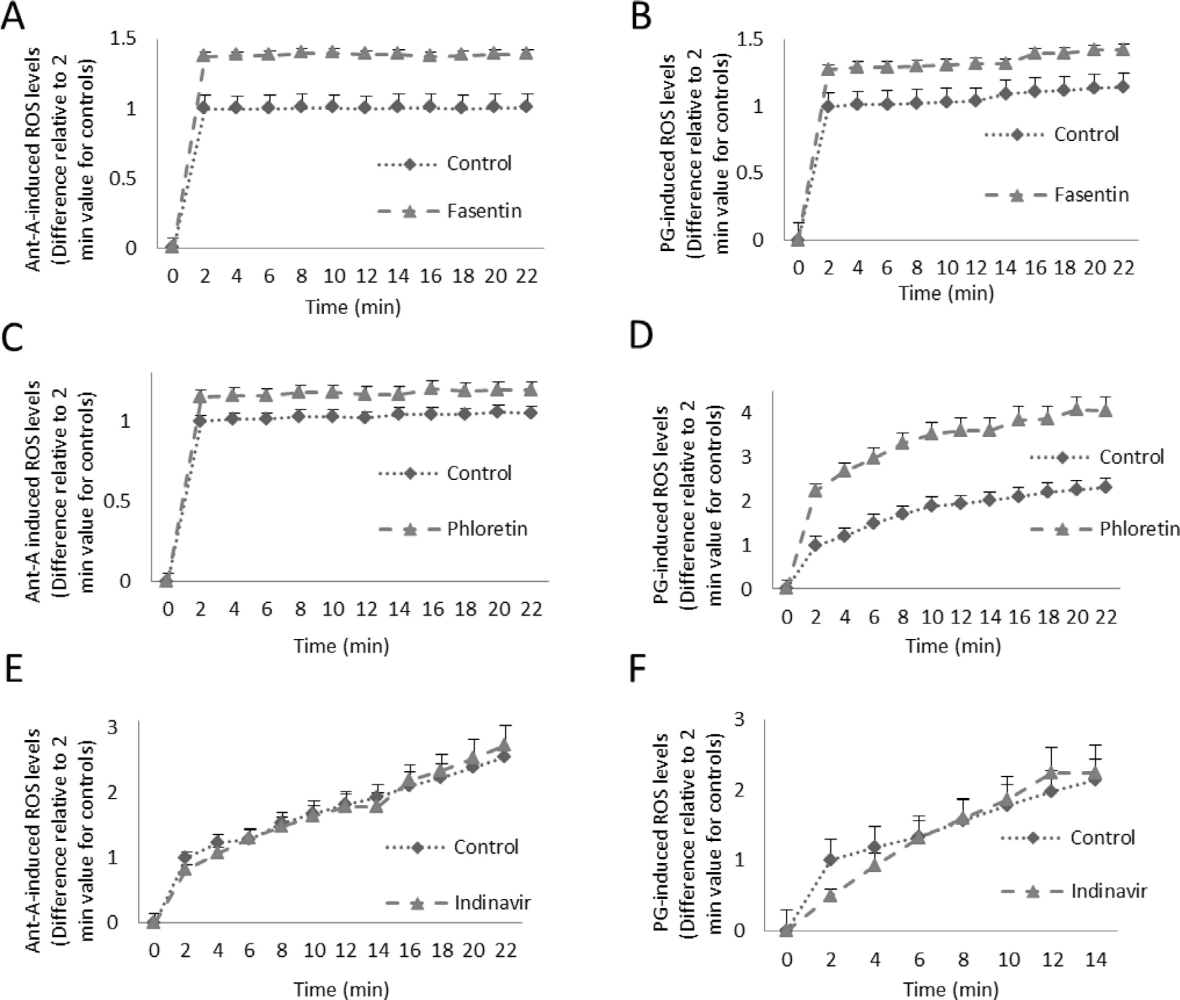
Inhibition of GLUT1 increases ROS levels. L6 myoblasts were subjected to a DCFDA ROS assay in the presence or absence of (A) GLUT1 inhibitor, 80 α/4M fasentin with induction of ROS by 100 α/4M antimycin A (AntA), (B) 80 α/4M fasentin with 50 α/4M pyrogallol (PG), a superoxide generator, (C) GLUT1 inhibitor, 100 α/4M phloretin, with induction of ROS by 100 α/4M AntA, (D) 100 α/4M phloretin with 50 α/4M PG, and (E) 100 α/4M indinavir, which inhibits GLUT4, with induction of ROS by Ant-A or (F) by PG. (*n* = 6/group. Differences are statistically significant for all time points after time 0, *p* < 0.05. Subpanels E and F do not display significant differences, except 1F at 2 min, *p* < 0.05).

The oxidative state of the cell is maintained by a balance between antioxidant and pro-oxidant levels. In cultured muscle cells, GLUT1 and GLUT4 transport glucose [23] and DHA [24], both of which can aid in antioxidant defense [11,13]. To determine the effects of GLUT1 inhibition on ROS levels, L6 myoblasts that had been loaded with DCFDA, a fluorescent ROS probe, were incubated in media plus and minus GLUT1 inhibitors, and then oxidative stress was induced by antimycin A (Ant-A) or pyrogallol (PG). GLUT1 inhibition with 80 α/4M fasentin increased both Ant-A- and PG-induced ROS levels (30•40%, *p* < 0.05) (Fig. 1A and B). GLUT1 inhibition with 100 α/4M phloretin raised Ant-A-induced ROS by 20% and PG-induced ROS levels by 2-fold (*p* < 0.05) (Fig. 1C and D). These data suggest that GLUT1 plays a role in the regulation of ROS levels.

To determine whether GLUT4 plays a role in regulation of ROS in muscle cells, DCFDA loaded L6 myoblasts were incubated in the presence or absence of 100 µM indinavir, which inhibits GLUT4 but not GLUT1, and then oxidative stress was induced by Ant-A or PG. From 4 min post-induction and beyond, indinavir treated muscles displayed no significant change in Ant-A or PG-induced ROS levels from control muscle cells (Fig. 1E and F). Thus, in muscle cells, GLUT4 does not play a role in regulation of ROS levels. Together, the data suggest that GLUT1 plays a more prominent role in regulation of ROS than does GLUT4.

### A carboxy terminus amino acid residue, GLUT1-S490, regulates ROS levels

GLUT1 has a cytosolic N-terminus, 12 membrane-spanning helices, and a cytosolic 42 amino acid C-terminus [25]. The c-terminus of GLUT1 contains a PDZ binding motif [26]. Removal of the last four amino acids which make up the GLUT1 PDZ binding motif results in GLUT1 being internalized from the plasma membrane [26]. Previous work has shown that GLUT1-S490, an ATM target within the PDZ binding motif, regulates GLUT1-mediated glucose and DHA transport [14]. For example, expression of GLUT1-S490A decreases glucose and DHA transport, cell surface localization of GLUT1, whereas GLUT1-S490D increases glucose and DHA transport and cell surface GLUT1. These alterations are concomitant with changes in association of the PDZ protein GIPC1 with GLUT1. This ATM target site of GLUT1, S490, is not present in GLUT4.

**Fig. 2:**
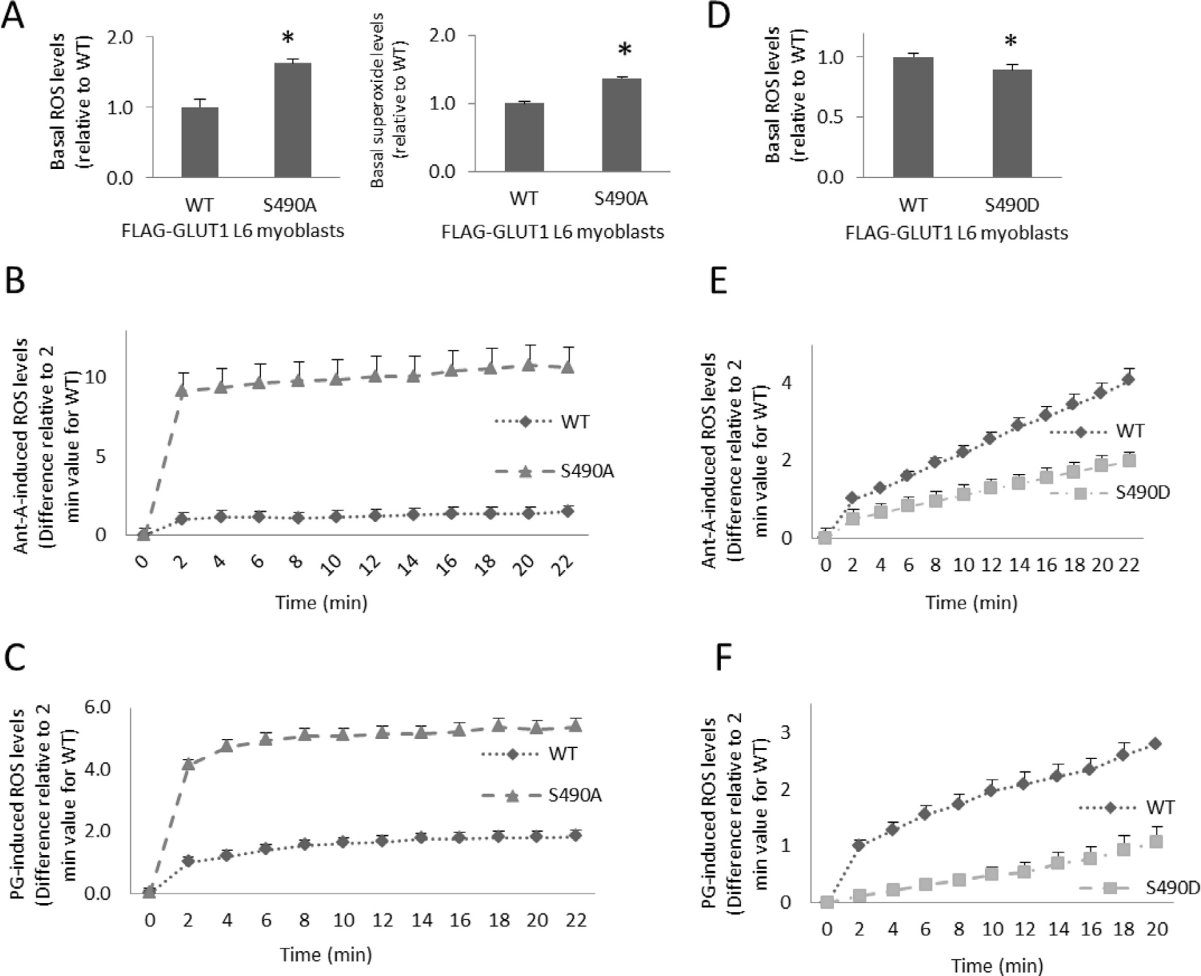
GLUT1-S490 mutations affect ROS levels. L6 myoblasts transiently transfected with FLAG-GLUT1 constructs or FLAG-GLUT1-S490 point mutation constructs were loaded with the general ROS fluorescent probe, DCFDA (for all data except the right panel of A), or a purported superoxide probe that accumulates in mitochondria, MitoSox Red (right panel of A only), and fluorescent readings were taken. (A) Cells expressing wild-type GLUT1 (WT) vs. GLUT1-S490A under basal conditions, (B) with 100 α/4M Ant-A induction of ROS or (C) with induction of ROS by 50 α/4M PG. (D) WT vs. GLUT1-S490D mutants under basal conditions, (E) with 100 α/4M Ant-A induction of ROS, or (F) with induction of ROS by 50 α/4M PG. (*n* = 6/group, *p* < 0.05 for all panels. Differences are statistically significant for all time points after time 0, *p* < 0.05).

To determine the role of GLUT1-S490 in regulation of ROS levels, L6 myoblasts transiently transfected with FLAG-GLUT1 constructs or FLAG-GLUT1 constructs containing a point mutation of GLUT-S490 were subjected to DCFDA ROS assays or in one experiment MitoSox-Red ROS assays. GLUT1-S490A mutants exhibited enhanced basal ROS levels as assessed with DCFDA (63%, *p* < 0.05) and enhanced basal Mitosox Red signal (25%, *p* < 0.05) (Fig. 2A). Fluorometric measures with Mitosox Red were not as robust as those with DCFDA, so all subsequent measures were with DCFDA. The Mitosox Red measures are listed as “superoxide levels” in panel A, although it must be acknowledged that Mitosox Red is by no means specific for superoxide, reacting, for example, with heme-containing proteins [27].

There was nearly a ten-fold increase in Ant-A-induced ROS levels (*p* < 0.05) (Fig. 2B), and a 5-fold increase in PG-induced ROS levels compared to controls (*p* < 0.05) (Fig. 2C). GLUT1-S490D mutants displayed diminished basal ROS levels (12%, *p* < 0.05) (Fig. 2D), and both Ant-A- and PG-induced ROS levels were lowered by roughly 50% in cells expressing GLUT1-S490D(*p* < 0.05) (Fig. 2E and F). These findings suggest that phosphorylation of GLUT1-S490 could play a role in decreasing oxidative stress.

### ATM may play a role in GLUT1-mediated ROS regulation

**Fig. 3:**
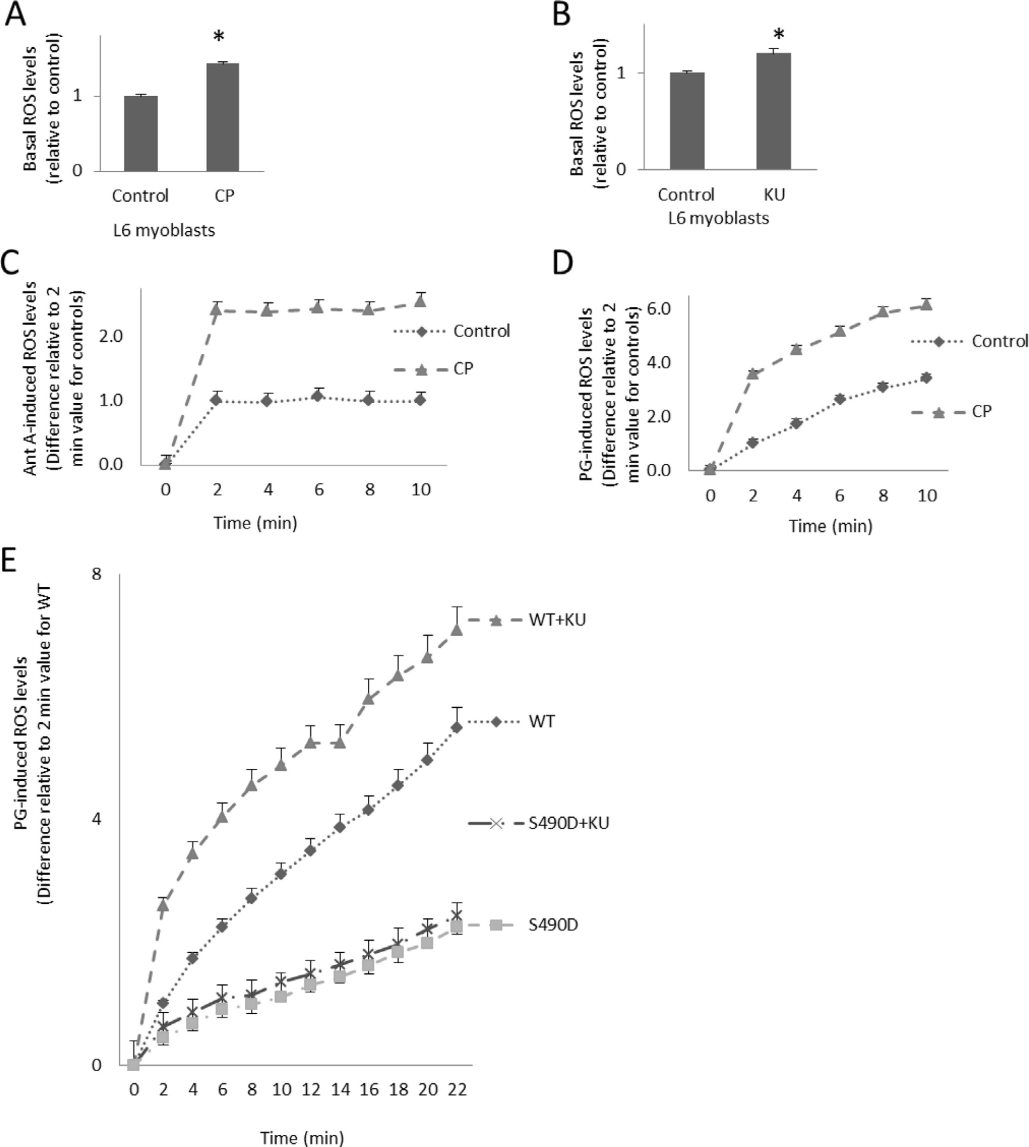
GLUT1-S490D prevents increases in ROS when ATM is inhibited. L6 myoblasts were subjected to a DCFDA ROS assay in the presence or absence of (A) ATM inhibitor, 6 α/4M CP466722 (CP), (B) ATM inhibitor, 10 α/4M KU55933 (KU), (C) 6 α/4M CP with 100 α/4M Ant-A induction of ROS, and (D) 6 α/4M CP with induction of ROS by 50 α/4M PG. (E) L6 myoblasts transiently transfected with FLAG-GLUT1 (WT) or FLAG-GLUT1-S490D (S490D) constructs were treated plus and minus 10 α/4M KU. (*n* = 6/group, ^*^p < 0.05 for all panels. Differences are statistically significant for all time points after time 0, *p* < 0.05, except for lines that visually overlap).

Previous work has shown that GLUT1-mediated glucose and DHA transport is regulated by ATM [14]. To decipher whether ATM inhibitors that were previously shown to lower GLUT1-mediated transport would increase ROS levels, as GLUT1 inhibition did (Fig. 1), L6 myoblasts were incubated in the presence and absence of ATM inhibitors and subjected to a DCFDA ROS assay. Basal ROS levels were elevated in L6 myoblasts exposed to ATM inhibitors, 6 α/4M CP and 10 α/4M KU, compared to controls (43% and 20%, respectively, *p* < 0.05) (Fig. 3A and B). ATM inhibition with 6 α/4M CP resulted in a 2-fold increase in Ant-A- and PG-induced ROS levels in L6 myoblasts (*p* < 0.05) (Fig. 3C and D).

To determine if these ATM effects on regulation of ROS levels might involve a direct effect on GLUT1, transiently transfected FLAG-GLUT1 L6 myoblasts expressing wild-type GLUT1 or GLUT1-S490A were loaded with DCFDA, incubated in media plus and minus 10 α/4M KU (an ATM inhibitor), and then exposed to superoxide generator, pyrogallol (50 α/4M). Cells expressing wild-type GLUT1 and treated with KU displayed a 28% increase in PG-induced ROS levels (*p* < 0.05, Fig. 3E). However, ATM inhibition with KU had no effect on cells expressing GLUT1-S490D (Fig. 3E). These findings suggest that ATM regulates ROS levels in a GLUT1-S490 dependent manner.

**Fig. 4:**
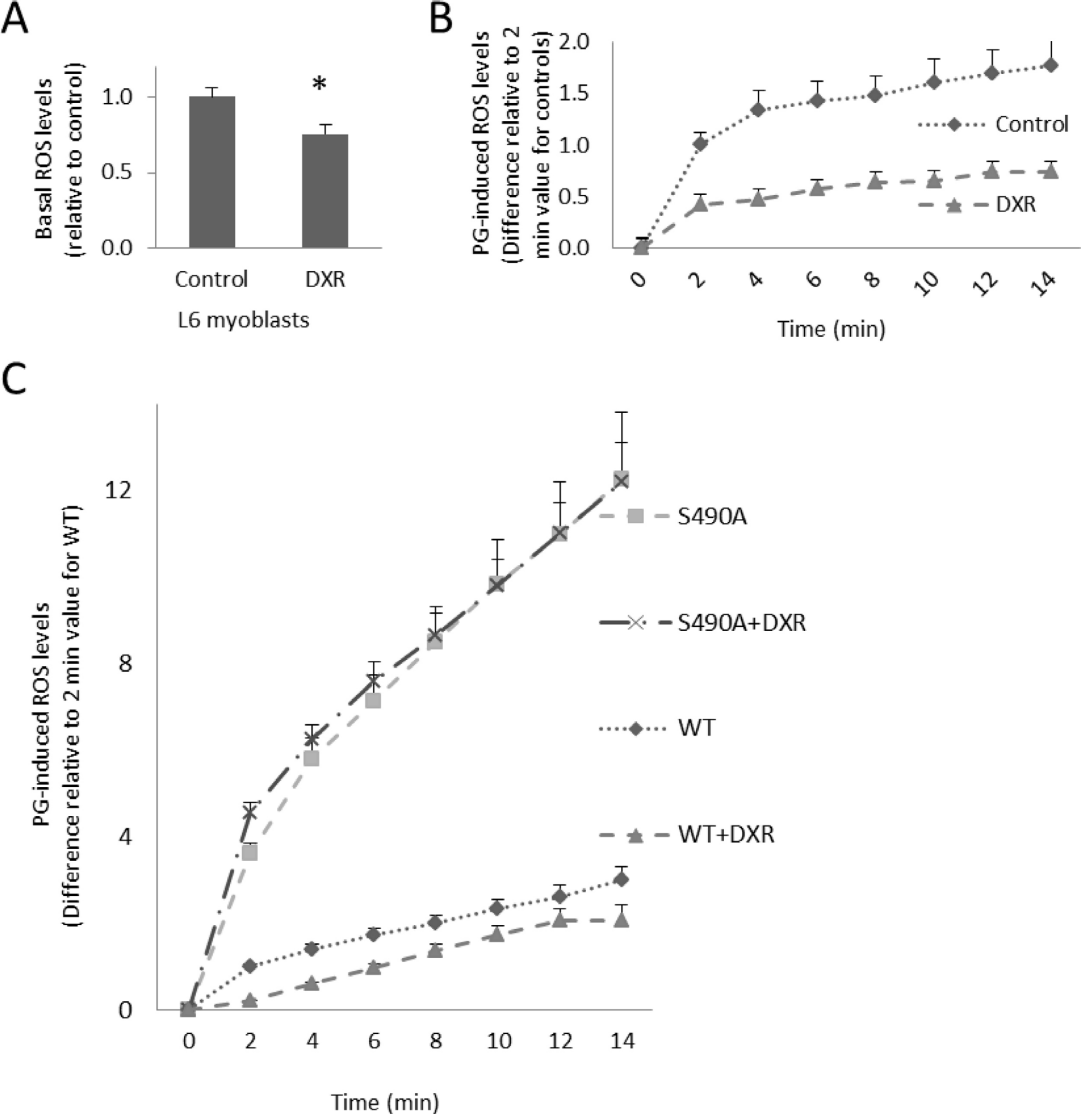
GLUT1-S490A prevents the ROS-lowering effect of doxorubicin. L6 myoblasts underwent a DCFDA ROS assay in the presence or absence of ATM activator, 1 α/4M doxorubicin (DXR), and fluorescent readings were taken (A) under basal conditions and (B) after exposure to PG. (C) Myoblasts expressing FLAG-GLUT1(WT) and FLAG-GLUT1-S490A (S490A) were treated plus and minus 1 α/4M DXR, an ATM activator. (*n* = 6/group, ^*^p < 0.05 for all panels. Differences are statistically significant for all time points after time 0, *p* < 0.05, except for lines that visually overlap).

Previous work has shown that ATM activation promotes GLUT1-mediated transport in skeletal muscles and cultured muscle cells [14]. To examine the role of ATM activation on ROS levels in muscle cells, a DCFDA ROS assay was performed after exposure of myoblasts to the ATM activator doxorubicin (DXR). L6 myoblasts incubated in 1 α/4M DXR displayed lowered basal and PG-induced ROS levels compared to vehicle-treated (0.1% DMSO) controls (25% and 65%, respectively, *p* < 0.05) (Fig. 4A and B). PG-induced ROS levels were markedly increased in cells transfected with GLUT1-S490A compared to cells transfected with wild-type GLUT1 (*p* < 0.05) (Fig. 4C). Furthermore, DXR had no effect on ROS levels in cells expressing GLUT-S490A (Fig. 4C). These data suggest that ATM activation could decrease ROS in a mechanism involving GLUT1-S490 phosphorylation.

### ROS and insulin action

**Fig. 5:**
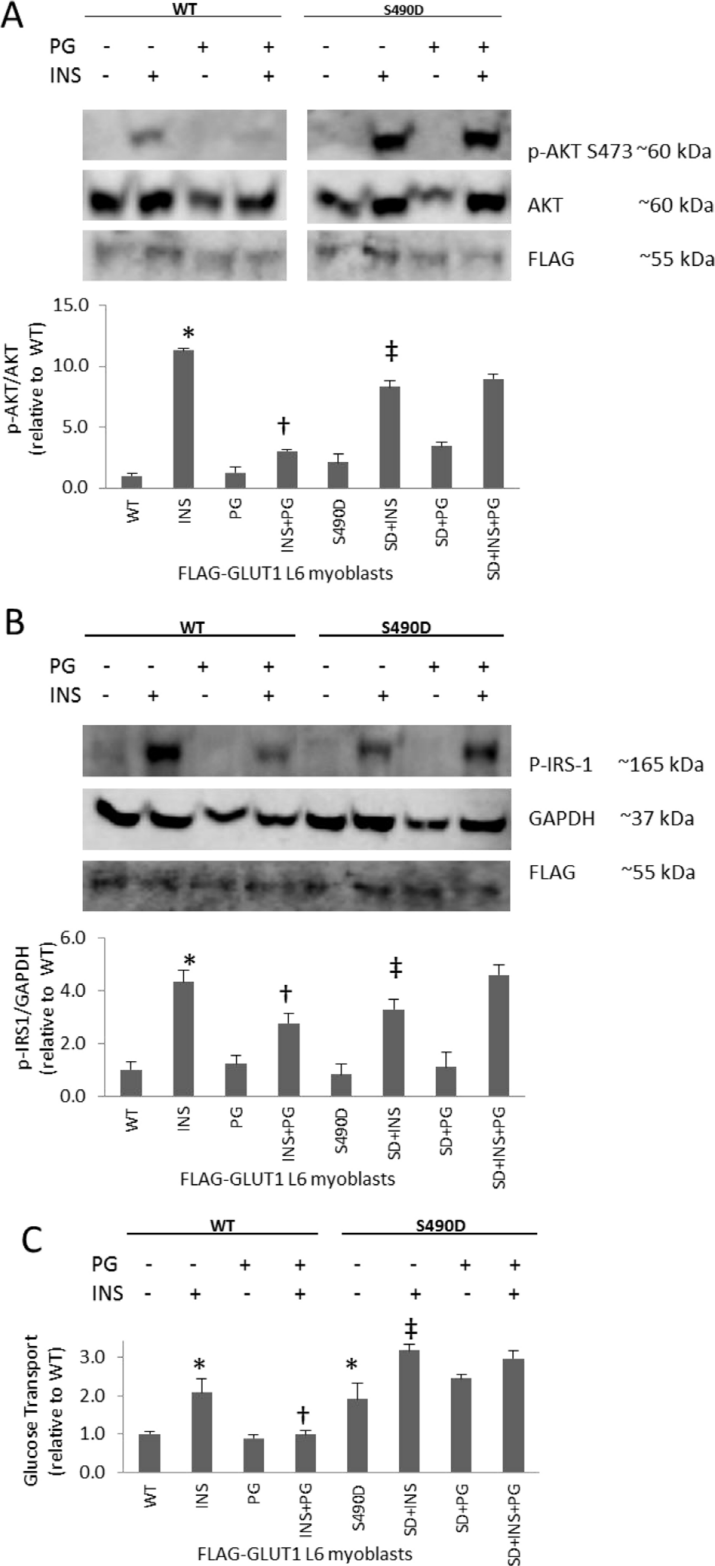
GLUT1-S490D prevents ROS-induced insulin resistance. L6 myoblasts transiently transfected with FLAG-GLUT1 (WT) constructs or FLAG-GLUT1-S490D (SD) point mutation constructs were treated plus and minus insulin (INS) and pyrogallol (PG), a superoxide generator, then subjected to (A) Western blot analysis probing for phosphorylated Akt S473 (p-Akt S473) and Akt as a loading control or (B) phosphorylated insulin receptor substrate 1 (p-IRS-1) and glyceraldehyde-3-phosphate dehydrogenase (GAPDH) as a loading control, and (C) a glucose transport assay (*n* = 3/group for the Western analysis and 6/group for the transport assay, ^*^p < 0.05 compared to WT, ^†^*p* < 0.05 compared to INS, and ^‡^*p*< 0.05 compared to S490D for all panels).

Elevated levels of hydrogen peroxide interfere with insulin action, as demonstrated by weakened insulin-stimulated GLUT4 translocation in 3T3-L1 adipocytes after long-term exposure to H_2_O_2_ [28]. Another study showed that ROS-induced insulin resistance caused by TNF-α and dexamethasone could be reversed by suppression of ROS via antioxidants and transgenes promoting antioxidant defense [29]. GLUT1-S490D has been shown to increase GLUT1-mediated glucose and DHA transport [14] and also to decrease ROS levels (Fig. 2B, D and F). To determine if GLUT1-S490D could prevent ROS-induced insulin resistance, L6 myoblasts transfected with FLAG-GLUT1 and FLAG-GLUT1-S490D mutants were incubated in media in the presence and absence of both insulin and PG then subjected to Western blot analysis exploring insulin signaling pathways. L6 myoblasts expressing FLAG-GLUT1 (wild type) treated with insulin displayed a 10-fold increase in phosphorylated Akt serine 473 (p-Akt S473) levels and a 4-fold rise in phosphorylated insulin receptor substrate 1 tyrosine 612 (p-IRS-1) compared to FLAG controls (*p* < 0.05) (Fig. 5A and B). However, the insulin-induced increase in p-Akt S473 was nearly fully prevented by exposure to PG (*p* < 0.05) (Fig. 5A). Likewise, the 4-fold escalation in p-IRS-1 caused by insulin was also diminished by PG (*p* < 0.05) (Fig. 5B). Thus, pyrogallol decreases insulin action in L6 myoblasts. Strikingly, expression of GLUT1-S490D prevented the PG-induced decreases in insulin-stimulated p-Akt S473 and p-IRS-1 levels (*p* < 0.05) (Fig. 5A and B). In accordance with these findings, PG decreased insulin-stimulated glucose transport in myoblasts transfected with the wild-type GLUT1 construct, but insulin action was normal in myoblasts transfected with the GLUT1-S490D construct (Fig. 5C). Expression of the GLUT1 transgenes was similar for wild-type GLUT1 and GLU1-S490D as demonstrated by the similar FLAG signal. Taken together, the data in Fig. 5 suggest that GLUT1-S490D prevents ROS-induced insulin resistance in L6 myoblasts.

## Discussion and conclusions

The findings presented in this study show that GLUT1 plays a role in regulation of ROS levels in muscle cells. For example, inhibition of GLUT1-but not inhibition of GLUT4-increased ROS levels caused by challenge with pyrogallol or antimycin A. Site mutation of GLUT1-S490, a known ATM phosphorylation target, to alanine (S490A) augmented ROS levels; in contrast, mutation of this ATM target site to aspartic acid (S490D) decreased ROS levels. Inhibition of ATM increased ROS, but not in cells expressing GLUT1 mutated to mimic phosphorylation at an ATM target site (S490D). Conversely, the ATM activator doxorubicin decreased ROS, but not in myoblasts expressing GLUT1-S490A. Finally, insulin resistance caused by a superoxide generator was prevented by expression of GLUT1-S490D. Together, the data suggest the novel idea that GLUT1 plays a role in antioxidant defense and also functions to maintain insulin action in the face of oxidative stress.

Insulin exerts its effects of lowering blood glucose levels by activating a signaling cascade that results in the translocation of GLUT4 [30]. IRS-1 undergoes tyrosine phosphorylation after insulin binding to the insulin receptor activates the receptor tm)s kinase activity [31]. Phosphorylation at Tyr 612 (corresponding to Y608 of murine IRS-1) and several other tyrosine residues of IRS-1 recruits other proteins to propagate the insulin signal [31]. Downstream of IRS-1, Akt mediates effects of insulin such as regulation of insulin-stimulated GLUT4 translocation [32]. Phosphorylation at S473 of Akt leads to activation of Akt kinase activity [33]. Here we show that GLUT1-S490D was able to rescue the ROS-induced decrease in insulin signaling at IRS-1 and Akt.

ATM is well known for its nuclear role of sensing and commencing the signaling for DNA damage and repair [34]. Double-stranded DNA breaks recruit and activate ATM which in turn coordinates a signaling cascade that solicits DNA repair machinery and cell cycle checkpoint proteins to repair the damaged DNA or initiate apoptosis [35]. Aside from ATM tm)s nuclear role, several studies have revealed that ATM displays cytosolic localizations [36,37], where ATM plays a role in ROS sensing and signaling [37]. ROS activate ATM via autophosphorylation of S1981 in a DNA damage-independent manner initiating a cytosolic signaling cascade that inhibits mTORC1 in an LKB1-AMPK-TSC2 dependent fashion [38]. Decreased mTOR activity is associated with decreased oxidative phosphorylation [39] which in turn lowers ROS. In an independent process, reactive oxygen species directly activate ATM, leading to subsequent activation of G6PDH and an increase in potential to reduce ROS [40]. Here we propose a novel way for ATM to reduce ROS via regulation of GLUT1.

Antioxidant defense proteins include single molecule antioxidants as well as intricate antioxidant enzyme systems [41]. Ascorbic acid [42], α-tocopherol (vitamin E) [43], β-carotene [44], and lipoic acid [45] are among the prominent small molecule antioxidants in the cell. The antioxidant enzyme systems include glutathione systems, superoxide dismutases, catalases, and peroxiredoxins [46,47]. The glutathione systems are composed of glutathione reductases, peroxidases, and S-tranferases, and have the ability to rapidly reduce DHA to ascorbate [48]. As an adaptive response, under immunological oxidative stress, hepatic endothelial cells display increased levels of GLUT1 and G6PDH [49]. G6PDH is the rate limiting enzyme in the pentose phosphate pathway [50], which produces NADPH [50]. All of the above listed antioxidant enzymes require NADPH directly or indirectly for functionality [49]. Through the increased activity of GLUT1 to bring in glucose to serve as a substrate (when converted to glucose-6-phosphate) for the pentose phosphate pathway to produce more NADPH, hepatic endothelial cells have developed a mechanism to reduce ROS and decrease oxidative stress [51]. Thus, GLUT1 expression has previously been shown to be associated with redox status. By use of GLUT1 inhibitors and site mutations that alter GLUT1-mediated transport, the current study has elevated GLUT1 from a factor associated with oxidative defense to a protein that plays a role in regulation of ROS levels.

In summary, a role of GLUT1 in regulation of ROS levels has been elucidated by means of GLUT1 inhibitors that increase ROS and expression of GLUT1 with mutations at an ATM target site that prevent (S490A) or mimic (S490D) phosphorylation and increase or decrease ROS, respectively. Notably, the ATM activator doxorubicin decreases ROS except in cells that express GLUT1-S490A, while inhibition of ATM increases ROS except in cells transfected with GLUT1S490D. Furthermore, expression of GLUT1-S490D protects cells against ROS-mediated insulin resistance. Thus, the findings suggest the novel idea that GLUT1 plays roles in regulation of ROS and maintenance of insulin action under conditions of oxidative stress.

## Conflicts of interest

No conflicts of interest exist for any of the authors.

## References

[1] Nohl H., Gille L., Staniek K. (2004). The mystery of reactive oxygen species derived from cell respiration. Acta Biochimica Polonica.

[2] Chambers M.A., Moylan J.S., Smith J.D., Goodyear L.J., Reid M.B. (2009). Stretch-stimulated glucose uptake in skeletal muscle is mediated by reactive oxygen species and p38 MAP-kinase. Journal of Physiology.

[3] Kang C., O tm)Moore K.M., Dickman J.R., Ji L.L. (2009). Exercise activation of muscle peroxisome proliferator-activated receptor-gamma coactivator-1alpha signaling is redox sensitive. Free Radical Biology and Medicine.

[4] Zembron-Lacny A., Naczk M., Gajewski M., Ostapiuk-Karolczuk J., Dziewiecka H., Kasperska A., Szyszka K. (2010). Changes of muscle-derived cytokines in relation to thiol redox status and reactive oxygen and nitrogen species. Physiological Research/Academia Scientiarum Bohemoslovaca.

[5] Furukawa S., Fujita T., Shimabukuro M., Iwaki M., Yamada Y., Nakajima Y., Nakayama O., Makishima M., Matsuda M., Shimomura I. (2004). Increased oxidative stress in obesity and its impact on metabolic syndrome. Journal of Clinical investigation.

[6] Evans J.L., Goldfine I.D., Maddux B.A., Grodsky G.M. (2003). Are oxidative stress-activated signaling pathways mediators of insulin resistance and beta-cell dysfunction?. Diabetes.

[7] Lockwood D.H., Amatruda J.M. (1983). Cellular alterations responsible for insulin resistance in obesity and type II diabetes mellitus. American Journal of Medicine.

[8] Anderson E.J., Lustig M.E., Boyle K.E., Woodlief T.L., Kane D.A., Lin C.T., Price J.W., Kang L., Rabinovitch P.S., Szeto H.H., Houmard J.A., Cortright R.N., Wasserman D.H., Neufer P.D. (2009). Mitochondrial H2O2 emission and cellular redox state link excess fat intake to insulin resistance in both rodents and humans. Journal of Clinical Investigation.

[9] Keller K., Mueckler M. (1990). Different mammalian facilitative glucose transporters expressed in Xenopus oocytes. Biomedica Biochimica Acta.

[10] Rumsey S.C., Kwon O., Xu G.W., Burant C.F., Simpson I., Levine M. (1997). Glucose transporter isoforms GLUT1 and GLUT3 transport dehydroascorbic acid. Journal of Biological Chemistry.

[11] Kruger N.J., von Schaewen A. (2003). The oxidative pentose phosphate pathway: structure and organisation. Current Opinion in Plant Biology.

[12] Hanukoglu I., Rapoport R. (1995). Routes and regulation of NADPH production in steroidogenic mitochondria. Endocrine Research.

[13] Savini I., Catani M.V., Duranti G., Ceci R., Sabatini S., Avigliano L. (2005). Vitamin C homeostasis in skeletal muscle cells. Free Radical Biology and Medicine.

[14] Andrisse S., Patel G.D., Chen J.E., Webber A.M., Spears L.D., Koehler R.M., Robinson-Hill R.M., Ching J.K., Jeong I., Fisher J.S. (2013). ATM and GLUT1-S490 phosphorylation regulate GLUT1 mediated transport in skeletal muscle. PloS One.

[15] Hickson I., Zhao Y., Richardson C.J., Green S.J., Martin N.M., Orr A.I., Reaper P.M., Jackson S.P., Curtin N.J., Smith G.C. (2004). Identification and characterization of a novel and specific inhibitor of the ataxia-telangiectasia mutated kinase ATM. Cancer Research.

[16] Rainey M.D., Charlton M.E., Stanton R.V., Kastan M.B. (2008). Transient inhibition of ATM kinase is sufficient to enhance cellular sensitivity to ionizing radiation. Cancer Research.

[17] Wieman H.L., Wofford J.A., Rathmell J.C. (2007). Cytokine stimulation promotes glucose uptake via phosphatidylinositol-3 kinase/Akt regulation of Glut1 activity and trafficking. Molecular Biology of the Cell.

[18] Bilan P.J., Mitsumoto Y., Maher F., Simpson I.A., Klip A. (1992). Detection of the GLUT3 facilitative glucose transporter in rat L6 muscle cells: regulation by cellular differentiation, insulin and insulin-like growth factor-I. Biochemical and Biophysical Research Communications.

[19] Ali S.F., LeBel C.P., Bondy S.C. (1992). Reactive oxygen species formation as a biomarker of methylmercury and trimethyltin neurotoxicity. Neurotoxicology.

[20] Lieven C.J., Hoegger M.J., Schlieve C.R., Levin L.A. (2006). Retinal ganglion cell axotomy induces an increase in intracellular superoxide anion. Investigative Ophthalmology and Visual Science.

[21] Dairaku N., Kato K., Honda K., Koike T., Iijima K., Imatani A., Sekine H., Ohara S., Matsui H., Shimosegawa T. (2004). Oligomycin and antimycin A prevent nitric oxide-induced apoptosis by blocking cytochrome C leakage. Journal of Laboratory and Clinical Medicine.

[22] Upadhyay G., Gupta S.P., Prakash O., Singh M.P. (2010). Pyrogallol-mediated toxicity and natural antioxidants: triumphs and pitfalls of preclinical findings and their translational limitations. Chemico-Biological Interactions.

[23] Klip A., Pâquet M.R. (1990). Glucose transport and glucose transporters in muscle and their metabolic regulation. Diabetes Care.

[24] Liang W.J., Johnson D., Jarvis S.M. (2001). Vitamin C transport systems of mammalian cells. Molecular Membrane Biology.

[25] Mueckler M., Makepeace C. (2009). Model of the exofacial substrate-binding site and helical folding of the human Glut1 glucose transporter based on scanning mutagenesis. Biochemistry.

[26] Wieman H.L., Horn S.R., Jacobs S.R., Altman B.J., Kornbluth S., Rathmell J.C. (2009). An essential role for the Glut1 PDZ-binding motif in growth factor regulation of Glut1 degradation and trafficking. Biochemical Journal.

[27] Zielonka J., Kalyanaraman B. (2010). Hydroethidine- and MitoSOX-derived red fluorescence is not a reliable indicator of intracellular superoxide formation: Another inconvenient truth. Free Radical Biology and Medicine.

[28] Rudich A., Tirosh A., Potashnik R., Hemi R., Kanety H., Bashan N. (1998). Prolonged oxidative stress impairs insulin-induced GLUT4 translocation in 3T3-L1 adipocytes. Diabetes.

[29] Houstis N., Rosen E.D., Lander E.S. (2006). Reactive oxygen species have a causal role in multiple forms of insulin resistance. Nature.

[30] Chang L., Chiang S.H., Saltiel A.R. (2004). Insulin signaling and the regulation of glucose transport. Molecular Medicine (Cambridge, Mass.).

[31] Hers I., Bell C.J., Poole A.W., Jiang D., Denton R.M., Schaefer E., Tavarèc) J.M. (2002). Reciprocal feedback regulation of insulin receptor and insulin receptor substrate tyrosine phosphorylation by phosphoinositide 3-kinase in primary adipocytes. Biochemical Journal.

[32] Hajduch E., Litherland G.J., Hundal H.S. (2001). Protein kinase B (PKB/Akt)•a key regulator of glucose transport?. FEBS Letters.

[33] Sarbassov D.D., Guertin D.A., Ali S.M., Sabatini D.M. (2005). Phosphorylation and regulation of Akt/PKB by the rictor-mTOR complex. Science (New York, N.Y.).

[34] Meyn M.S. (1995). Ataxia-telangiectasia and cellular responses to DNA damage. Cancer Research.

[35] Kastan M.B., Bartek J. (2004). Cell-cycle checkpoints and cancer. Nature.

[36] Watters D., Kedar P., Spring K., Bjorkman J., Chen P., Gatei M., Birrell G., Garrone B., Srinivasa P., Crane D.I., Lavin M.F. (1999). Localization of a portion of extranuclear ATM to peroxisomes. Journal of Biological Chemistry.

[37] Alexander A., Walker C.L. (2010). Differential localization of ATM is correlated with activation of distinct downstream signaling pathways. Cell Cycle (Georgetown, Tex.).

[38] Alexander A., Cai S.L., Kim J., Nanez A., Sahin M., MacLean K.H., Inoki K., Guan K.L., Shen J., Person M.D., Kusewitt D., Mills G.B., Kastan M.B., Walker C.L. (2010). ATM signals to TSC2 in the cytoplasm to regulate mTORC1 in response to ROS. Proceedings of the National Academy of Sciences of the United States of America.

[39] Halicka H.D., Zhao H., Li J., Lee Y.S., Hsieh T.C., Wu J.M., Darzynkiewicz Z. (2012). Potential anti-aging agents suppress the level of constitutive mTOR- and DNA damage-signaling. Aging.

[40] Cosentino C., Grieco D., Costanzo V. (2011). ATM activates the pentose phosphate pathway promoting anti-oxidant defence and DNA repair. EMBO Journal.

[41] Ji L.L. (2007). Antioxidant signaling in skeletal muscle:a brief review. Experimental Gerontology.

[42] May J.M. (1998). Ascorbate function and metabolism in the human erythrocyte. Frontiers in Bioscience: a Journal and Virtual Library.

[43] Weber F., Gloor U., Wñ/4rsch J., Wiss O. (1964). Studies on the absorption, distribution and metabolism of 1-alpha-tocopherol in the rat. Biochemical and Biophysical Research Communications.

[44] Gillman J., Norton K.B., Rivett D.E., Sutton D.A. (1956). The identification and determination of vitamin A and beta-carotene in an animal-fat mixture. Biochemical Journal.

[45] Tsunoda J.N., Yasunobu K.T. (1967). Mammalian lipoic acid activating enzyme. Archives of Biochemistry and Biophysics.

[46] Rhee S.G., Kim K.H., Chae H.Z., Yim M.B., Uchida K., Netto L.E., Stadtman E.R. (1994). Antioxidant defense mechanisms: A new thiol-specific antioxidant enzyme. Annals of the New York Academy of Sciences.

[47] Rhee S.G., Chae H.Z., Kim K. (2005). Peroxiredoxins:a historical overview and speculative preview of novel mechanisms and emerging concepts in cell signaling. Free Radical Biologyand Medicine.

[48] Linster C.L., Van Schaftingen E. (2007). Vitamin C. Biosynthesis, recycling and degradation in mammals. FEBS Journal.

[49] Spolarics Z. (1998). Endotoxemia, pentose cycle, and the oxidant/antioxidant balance in the hepatic sinusoid. Journal of Leukocyte Biology.

[50] Prostko C.R., Fritz R.S., Kletzien R.F. (1989). Nutritional regulation of hepatic glucose-6-phosphate dehydrogenase. Transient activation of transcription. Biochemical Journal.

[51] Spolarics Z. (1996). Endotoxin stimulates gene expression of ROS-eliminating pathways in rat hepatic endothelial and Kupffer cells. American Journal of Physiology.

